# The Prevalence and Characteristics of Infective Endocarditis in Liver Transplant Recipients: Insights From National Inpatient Sample Database

**DOI:** 10.1002/clc.70130

**Published:** 2025-04-14

**Authors:** Ajit Brar, Ayushi Garg, Isha Kohli, Soumiya Ravi, Carol Singh, Aalam Sohal, M Luay Alkotob

**Affiliations:** ^1^ Department of Internal Medicine Hurley Medical Center Flint Michigan USA; ^2^ Bloomberg School of Public Health Johns Hopkins University Baltimore Maryland USA; ^3^ Trident Medical Center North Charleston South Carolina USA; ^4^ Graduate Program in Public Health, Icahn School of Medicine, Mount Sinai New York New York USA; ^5^ Department of Internal Medicine University of Arizona Tucson Arizona USA; ^6^ Dayanand Medical College and Hospital Ludhiana India; ^7^ Department of Hepatology Liver Institute Northwest Seattle Washington USA; ^8^ Department of Internal Medicine, Division of Cardiology Michigan State University at Hurley Medical Center Flint Michigan USA

**Keywords:** database, infective endocarditis, liver transplant, national inpatient sample, staphylococcus, transplant

## Abstract

**Background:**

Liver transplant (LT) recipients are immunocompromised and thus predisposed to various bacterial and fungal infections, including infective endocarditis (IE). The current paper aims to determine the prevalence, characteristics, and outcomes of IE in LT recipients.

**Methods:**

The National Inpatient Sample (NIS) data from 2016 to 2020 was used to identify LT recipients. Patients were separated into two groups based on the presence of IE. Information was collected on patient demographics, hospital characteristics, infections, comorbidities, and outcomes. Multivariate logistic regression was performed to assess the impact of IE on outcomes after adjusting for confounding factors.

**Results:**

A total of 170 650 patients who underwent LT were identified using NIS data from 2016 to 2020, of which 0.003% had IE. IE group had higher odds of in‐hospital mortality [aOR 2.2 (95% CI 1.07–4.78)], Shock [aOR 2.7 (95% CI 1.61–4.65)], ICU admission [aOR 2.40 (95% CI 1.4–4.2)], longer Length of Stay [adj. Coeff‐ 3.4 days (95% CI −0.89–5.9, *p* < 0.008)], and higher hospitalization charges (adj. coeff‐$65271.52, 95% CI $14 825–$115 718) than LT without IE group.

**Conclusion:**

Staphylococcus was present in 18.6% of IE in LT, followed by enterococcus (12.8%) and gram‐negative bacteria (9.8%). Concomitant IE was associated with increased in‐hospital death, ICU stay, and shock. The IE group was also associated with increased LOS and total charges compared to the LT without IE. Although the prevalence of IE is low in LT recipients, its presence portends worse outcomes.

## Graphical abstract

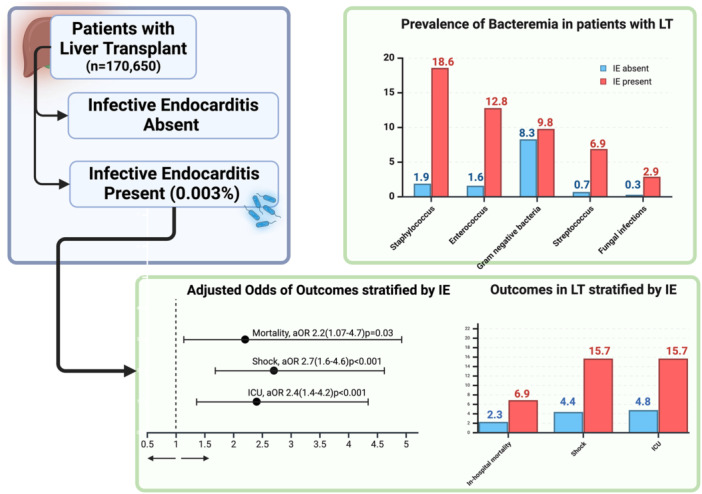

**Abbreviations**
AKI: Acute kidney injury
ESLD: End Stage Liver Disease
ICU: Intensive care unit
IE: Infective Endocarditis
IVDU: Intravenous drug use
LOS: Length of stay
LT: Liver transplant
NIS: National inpatient sample
SOT: Solid organ transplant

## 1 Introduction

LT is the only curative treatment available for patients with End‐stage liver disease (ESLD) [1]. Infections and Cardiovascular disease are leading causes of mortality in patients who have undergone Liver transplantation [2, 3]. Impairment of the immune system due to ESLD predisposes these patients to various bacterial and fungal infections [4, 5, 6, 7]. As a result, liver disease is associated with a higher incidence of IE than individuals without liver disease [8]. Furthermore, IE and bacteremia in the setting of immunosuppression correlate with higher mortality [9]. While much is known regarding the prevalence and outcomes of IE in patients with ESLD, similar data regarding outcomes or characteristics in LT recipients is currently sparse.

A significant amount of data on the subject of IE in LT stems from case reports and small case series. To summarize the current literature, Ioannou et al. performed a meta‐analysis of 39 studies and 62 liver transplant recipients [10]. In their study, Loannou et al. observed a mortality rate of 43.5%. Furthermore, 69.4% of mortality was attributed to gram‐positive bacteria, followed by 25.8% to fungal infections [10]. On the other hand, another study of 14 cases of IE in liver and kidney transplant recipients reported mortality to be as low as 4.6%. No nationwide study has comprehensively reported the prevalence, causative organisms, and outcomes of IE in LT recipients [11]. In this study, we used the NIS to assess the prevalence and impact of IE on outcomes in liver transplant recipients (LTR).

## 2 Patients and Methods

### 2.1 Data Source

The National Inpatient Sample (NIS), maintained by the Healthcare Cost and Utilization Project (HCUP), is the largest database of inpatient hospital stays in the United States. It contains information on 35 million weighted hospitalizations annually. Information regarding this data source has been discussed in previous studies. Each hospitalization is deidentified and maintained in the NIS as a unique entry with one primary discharge diagnosis and up to 39 secondary diagnoses during that hospitalization, depending on the year of data collection. Each entry carries patient demographics, including age, sex, race, insurance status, primary and secondary procedures (up to 25), hospitalization outcome, total charges, and LOS. IRB approval was not required as this study was conducted on publicly available deidentified data. It collects data from a 20% stratified sample of hospitals in 37 states in the United States and has been reliably used to estimate disease burden and outcomes. NIS contains data on 7 million unweighted and 35 million weighted hospitalizations annually. Each hospitalization is deidentified and maintained in the NIS as a unique entry with one primary discharge diagnosis and up to 39 secondary diagnoses. Each entry carries patient demographics, including age, sex, race, insurance status, primary and secondary procedures (up to 25), hospitalization outcome, total charges, and LOS. IRB approval was not required as it is publicly available deidentified data.

### 2.2 Study Population

**FIGURE 1:**
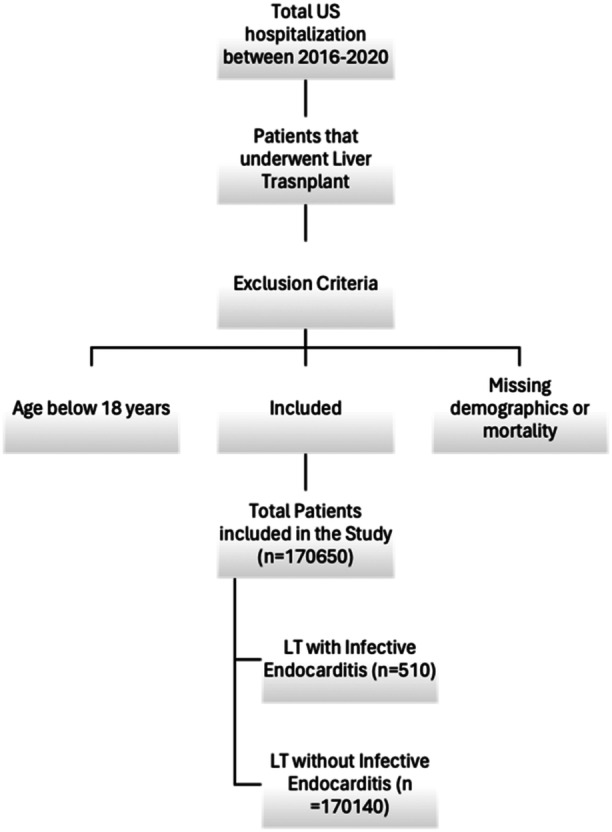
Inclusion flow diagram of the study population.

Patients with an index diagnosis of LT were identified using International Classification of diseases‐10th revision (ICD‐10) codes from the National Inpatient Sample (NIS) 2016–2020. We excluded patients with missing demographics/mortality, age < 18 years, or a history of other organ transplants. 170 650 patients were included in the study. The patients were stratified into two groups based on the absence or presence of IE, as presented in Figure 1.

### 2.3 Study Variables

Data was collected regarding patient demographics such as age, gender, race, insurance status, income quartile, and hospital characteristics (such as region, bed size, location, and academic status). We also further assessed Charlson comorbidities using the Charlson‐Deyo Comorbidity Index. This is a well‐validated index based on ICD 10‐CM codes meant to be used in large administrative data to predict mortality and hospital resource use. Data regarding decompensations of liver disease was also collected. We also collected data on common infectious causes such as staphylococcus, streptococcus, enterococcus, gram‐negative bacteria, and fungal infection.

### 2.4 Study Outcomes

The primary outcome of the study was in‐hospital mortality. Secondary outcomes included shock, intensive care unit (ICU), acute kidney injury (AKI), length of stay (LOS), and total hospitalization charges (THC). A patient was considered to have ICU admission if they required mechanical ventilation or vasopressor use.

### 2.5 Statistical Analysis

National estimates were generated using discharge weights provided by NIS. Categorical variables were compared using the chi‐square, whereas an independent sample *t*‐test was used for continuous variables. Univariate analysis was done to identify relationships between the study variables and outcomes. To assess the impact of IE on outcomes, multivariate logistic and linear regression analysis was performed by including only those variables that were noted to have *p* < 0.1 on univariate analysis. A two‐sided *p* < 0.05 indicates statistical significance, and the result was reported as adjusted ORs (aORs) for categorical variables and adjusted coefficients (adj coeff.) with 95% confidence intervals (CIs) (Tables 1, 2, 3).

**TABLE 1:** Patient demographics and hospital characteristics stratified by development of infective endocarditis.

Demographics	Absence of IE *n* (%)	Presence of IE *n* (%)	*p* value
**Age category**			0.09
18‐44	2250 (13.1)	35 (6.9)	
45‐64	75 615 (44.4)	215 (42.2)	
> 65	72 275 (42.5)	260 (51)	
**Sex**			0.03
Male	102 480 (60.2)	360 (70.6)	
Female	67 660 (39.8)	150 (29.4)	
**Race**			0.29
White	120 155 (70.6)	365 (71.6)	
African American	15 605 (9.2)	75 (14.7)	
Hispanic	23 570 (13.9)	55 (10.8)	
Asian/Pacific Islander	4645 (2.7)	**	
Native American	1215 (0.71)	0 (0)	
Other	4950 (2.90)	10 (1.9)	
**Primary expected payer**			0.51
Medicare	104 170 (61.2)	360 (70.6)	
Medicaid	17 680 (10.4)	50 (9.8)	
Private insurance	43 380 (25.5)	95 (18.6)	
Uninsured	1605 (0.9)	0	
**Median household income**			0.74
Lowest quartile	43 965 (25.8)	140 (27.5)	
Second quartile	44 470 (26.1)	130 (25.5)	
Third quartile	44 650 (26.2)	150 (29.4)	
Fourth quartile	37 055 (21.8)	90 (17.7)	
**Region of hospital**			0.36
Northeast	33 600 (19.8)	85 (16.7)	
Midwest	37 035 (21.8)	140 (27.5)	
South	65 905 (38.7)	170 (33.3)	
West	33 600 (19.8)	115 (22.6)	
**Location of the hospital**			0.50
Rural location	7315 (4.3)	15 (2.9)	
Urban location	162 825 (95.7)	495 (97.1)	
**Teaching status of the hospital**			0.85
Non teaching hospitals	3485 (17.9)	95 (18.6)	
Teaching hospitals	139 655 (82.1)	415 (81.4)	
**Bed size of hospital**			0.71
Small	21 385 (12.6)	55 (10.8)	
Medium	3305 (22.5)	130 (25.5)	
Large	110 450 (64.9)	325 (63.7)	

**TABLE 2:** Underlying comorbidities seen in patients stratified by development of infective endocarditis.

	Absence of IE *n* (%)	Presence of IE *n* (%)	*p* value
Prosthetic heart valve	2330 (1.4)	25 (4.9)	0.002
Acute myocardial infarction	14 040 (8.3)	60 (11.8)	0.198
Congestive heart failure	34 545 (20.3)	200 (39.2)	< 0.001
Peripheral vascular disease	9645 (5.7)	65 (12.8)	0.002
Cerebrovascular disease	9065 (5.3)	80 (15.7)	< 0.001
Dementia	4045 (2.4)	15 (2.9)	0.709
Chronic obstructive pulmonary disease	29 385 (17.3)	105 (20.6)	0.366
Rheumatoid disease	3295 (1.9)	**	0.481
Peptic ulcer disease	2855 (1.7)	10 (2)	0.824
Mild liver disease	157 350 (92.5)	480 (94.1)	0.531
Diabetes without complications	34 680 (20.4)	130 (25.5)	0.199
Diabetes with complications	51 620 (30.3)	180 (35.3)	0.265
Hemiplegia or paraplegia	2240 (1.3)	5 (1)	0.765
Renal disease	97 295 (57.2)	360 (70.6)	0.005
Cancer	7995 (4.7)	20 (3.9)	0.71
Moderate to severe liver disease	15 930 (9.4)	35 (6.9)	0.387
Metastatic cancer	4740 (2.8)	15 (2.9)	0.923
AIDS	415 (0.2)	0	0.64
Hyperlipidemia	41 950 (24.7)	130 (25.5)	0.846
Smoking	60 790 (35.7)	200 (39.2)	0.472
Alcohol abuse	15 440 (9.1)	20 (3.9)	0.07
Obesity	20 000 (11.7)	55 (10.8)	0.759
ALD	6400 (3.8)	10 (2)	0.339
Hepatitis B	3330 (2)	15 (2.9)	0.473
Hepatitis C	16 320 (9.6)	55 (10.8)	0.68
NASH	4970 (2.9)	0	0.08
Hepatocellular carcinoma	1445 (0.8)	10 (2)	0.219
Ascites	11 640 (6.8)	45 (8.8)	0.429
Varices	580 (0.3)	0	0.55
Spontaneous bacterial peritonitis	820 (0.5)	15 (2.9)	< 0.001
Hepatorenal syndrome	1660 (1)	0	0.322
Hepatic encephalopathy	10 805 (6.4)	60 (11.8)	0.026

*Note:* Values < 10 were not reported as per HCUP policy.

**TABLE 3:** Bacterial infections, stratified by the presence of infective endocarditis.

	Absence of IE *n* (%)	Presence of IE *n* (%)	*p*‐value
Staphylococcus	3195 (1.9)	95 (18.6)	< 0.001
Enterococcus	2665 (1.6)	65 (12.8)	< 0.001
Gram negative bacteria	14 085 (8.3)	50 (9.8)	0.57
Streptococcus	1270 (0.7)	35 (6.9)	< 0.001
Fungal infections	540 (0.3)	15 (2.9)	< 0.001

## 3 Results

### 3.1 Baseline Characteristics

**FIGURE 2:**
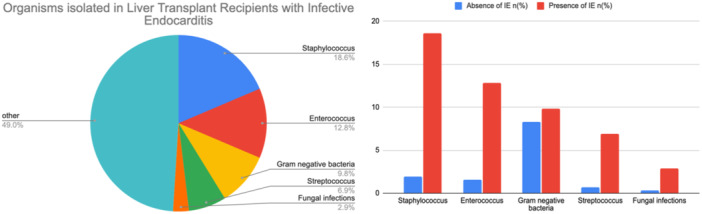
(a) Organisms isolated in LT with IE (b) Bacterial infections stratified by the presence of IE.

A total of 170 650 patients who underwent LT were identified using NIS data 2016–2020, of which 0.003% had IE. In the IE group, males represented 70.6%, 71.6% were Caucasians and 70.6% had medicare insurance. The IE with LT group had a higher prevalence of congestive heart failure (39.2% vs. 20.9%), peripheral vascular disease (12.8% vs. 5.7%), renal disease(70.6% vs. 57.2%), cerebrovascular disease(15.7% vs. 5.3%) prosthetic heart valve (4.9% vs. 1.4%) Spontaneous bacterial peritonitis (2.9% vs. 0.5%) and hepatic encephalopathy (11.8% vs. 6.4%). In the IE with LT group, the most common causative organisms were staphylococcus (18.6%), enterococcus (12.8%), gram‐negative bacteria (9.8%), and streptococcus (6.9%), as noted in Figure 2a. A complete list of demographic characteristics is presented in Supporting Information S1: Table SI, comorbidities in Supporting Information S1: Table SII, and Organisms identified in Supporting Information S1: Table SIII and Figure S2b.

**FIGURE 3:**
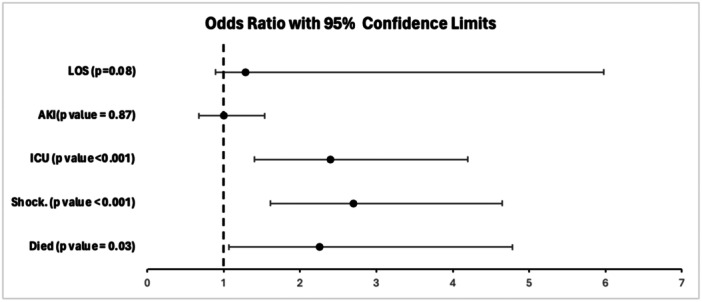
Adjusted odds ratio stratified by the presence of infective endocarditis.

**FIGURE 4:**
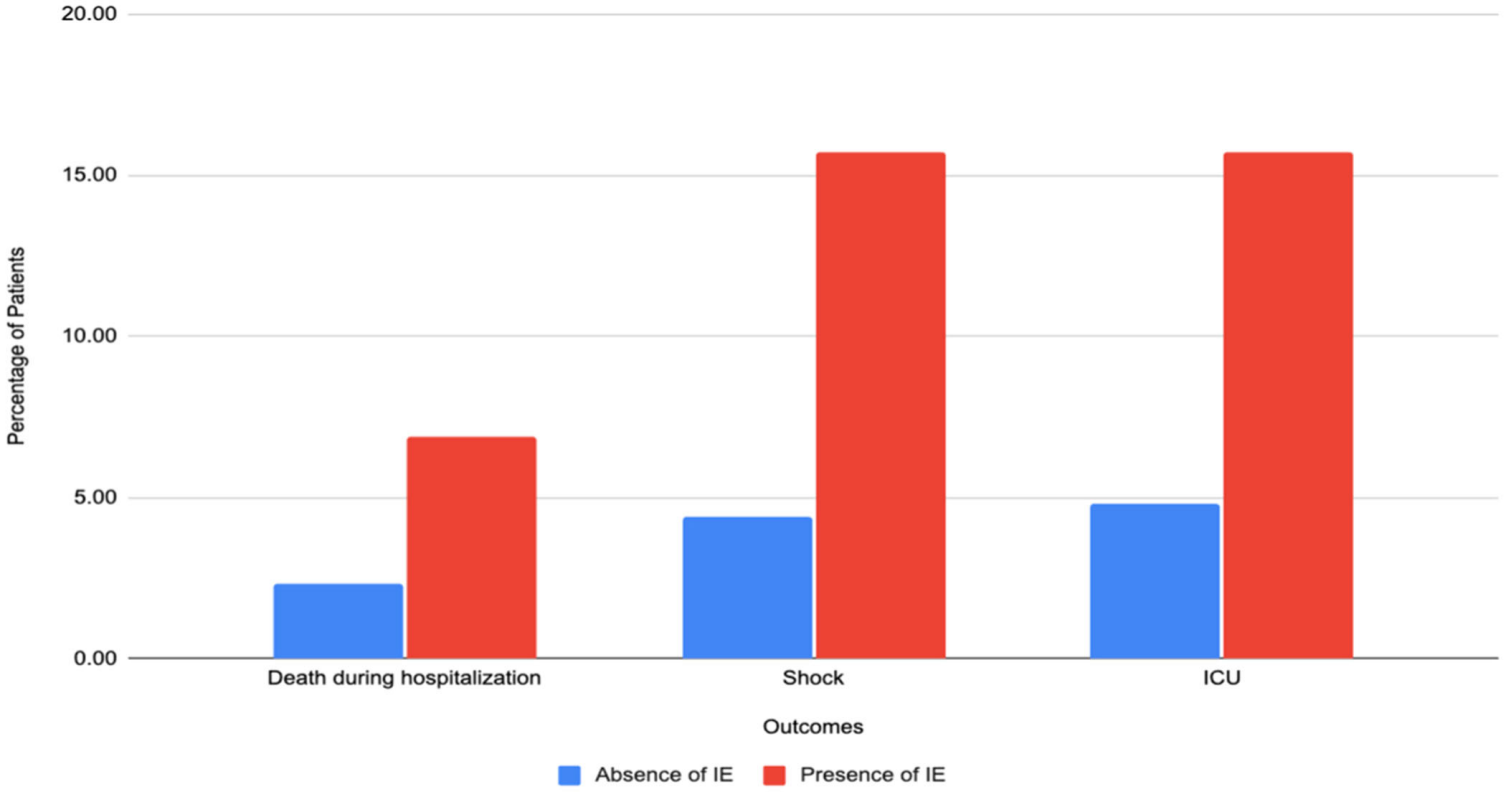
Outcomes stratified by the presence of infective endocarditis.

### 3.2 Outcomes

1. **In‐hospital mortality‐** The presence of IE was associated with a significantly higher mortality rate of 6.9% compared to 2.3% in LT without IE. Multivariate regression model revealed IE in the LT group had increased odds of mortality [aOR 2.2 (95% CI 1.07–4.78, *p* = 0.03)] as noted in Figures 3 and 4.
2. **Shock‐** The presence of IE was associated with a significantly higher rate of shock of 15.7% compared to 4.4% in LT without IE. Multivariate regression model revealed IE in the LT group had increased odds of shock [aOR 2.7 (95% CI 1.61–4.65, *p* < 0.001)]
3. **ICU admissions‐** The presence of IE was associated with a significantly higher rate of ICU admission of 15.7% compared to 4.8% in LT without IE. Multivariate regression model revealed IE in the LT group had increased odds of ICU admission [aOR 2.4 (95% CI 1.4–4.2, *p* < 0.001)]
4. **Acute Kidney Injury‐** The presence of IE was associated with a significantly higher rate of AKI of 42.2% compared to 38.2% in LT without IE. The multivariate regression model revealed no significant difference between LT with and without IE groups.
5. **Length of stay‐** The presence of IE was associated with a significantly longer length of stay of 10.6 days compared to 5.6 days in LT without IE. The multivariate regression model revealed IE in the LT group had increased odds of shock [adj. Coeff‐ 3.4 days (95% CI 0.89–5.9, *p* < 0.008)].
6. **Total hospitalization charges‐** The presence of IE was associated with significantly higher hospitalization charges of $154 807 compared to $67 997 in LT without IE. The multivariate regression model revealed IE in the LT group had increased odds of shock (adj. Coeff.‐ $65 271, 95% CI‐$14 825–$115 718, *p*‐0.01).

## 4 Discussion

The aim of our study was to assess the prevalence of IE in LT in the United States using the national data of hospitalized patients and the most common causative organisms identified. To our knowledge, this is the first national database study to compare the difference between outcomes in LT recipients with and without IE. Our study noted the frequency of IE in LT to be 0.3%. This is slightly lower than a study by Paterson et al. that reported the incidence of IE in LT recipients to be 1.7% in a multicenter study [12]. The incidence in our study is likely lower as this was a cross‐sectional and did not follow patients over multiple hospitalizations. On the other hand, SOT accounts for 1.6%–1.8% of all IE cases. Baral et al. identified SOT in 1.6% of IE cases through the NIS database. However, their study only studied 9 LT patients [13]. Similarly, Martinez‐Selles et al. observed that 1.8% of patients had SOT with IE [11]. and their study included 18 liver transplant recipients. In summary, most data on SOT involve relatively low numbers of LT. Our study is one of the largest studies reporting outcomes in 510 liver transplant recipients diagnosed with IE.

The IE with LT subgroup in our study consisted of 29.4% females. This may be attributed to the ability of estrogen to protect against epithelial damage and mount a stronger response to bacteremia, as demonstrated in various animal models [14] [15, 16]. The IE with LT subgroup demonstrated a higher prevalence of comorbidities, including CHF, PVD, renal disease, CVA, SBP, and hepatic encephalopathy. Furthermore, IE in the LT group had a higher prevalence of prosthetic valves. The higher prevalence of SBP and hepatic encephalopathy in the IE with LT group may be attributed to a predisposition to infections due to decreased protein and immunoglobulin synthesis in ESLD patients [17].

Our study identified causative organisms in 51% of cases of LT with IE. Staphylococcus accounted for 18.6% of all cases, followed by 12.8% enterococcus, 9.8% gram‐negative bacteria, 6.9% streptococcus, and 2.9% fungal (Figure 2a). This is in agreement with the findings of Eichenberger et al. who observed staphylococcal was the most common cause of SOT with IE [18]. Interestingly, the incidence of gram‐negative infection in the LT with IE was higher than that of streptococci (Figure 2b). Moreover, the incidence of gram‐negative bacteria noted in our study is similar to the 9.7% reported by Ioannau et al. [10]. The prevalence of fungal infections in the IE group was 2%. Although rare, fungal endocarditis portends higher mortality and adverse outcomes [19].

LT with IE may be linked with a higher mortality rate in comparison with SOT with IE. A meta‐analysis by Ionnau et al. observed a mortality rate of 43.5% in LT with IE [10]. However, these results should be read cautiously as this meta‐analysis of 62 patients included 28 case reports, which may have confounded the results. In our study, LT with IE exhibited an in‐hospital mortality rate of 6.9% as well as a higher odds of death in comparison with LT without IE (Figure 3). Additionally, this is higher than the 4.6% mortality rate highlighted by Baral et al. in the SOT with IE group [13]. Additionally, our study presented significantly higher rates of shock and ICU admissions in LT with IE group (Figure 4). Finally, a significantly higher LOS and total charges were observed in LT with the IE group. These may be attributed to higher disease severity or the need for further investigation, such as transesophageal echocardiogram or even valvular surgery. This highlights the need to identify these patients earlier during the disease course, thereby preventing IE‐related complications (Central Illustration 1).

**Central_Illustration 1:**
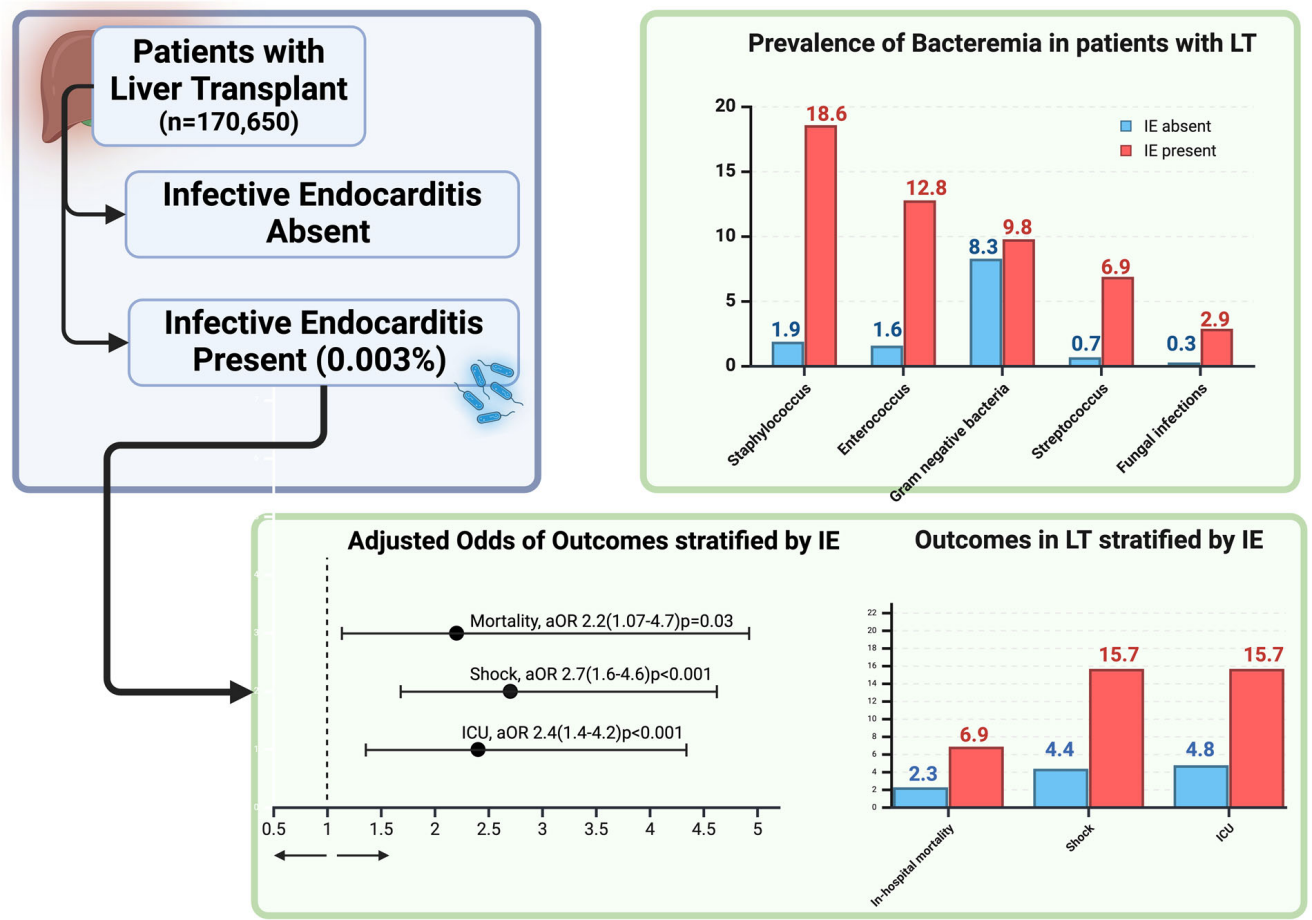
The impact of Infective Endocarditis in patients with Liver Transplant needs further inquiry. Analysis of 170 650 patients with LT based on the presence of infective endocarditis was performed. Staphylococcus (18.6%) and Enterococcus (12.8%) were the most common strains identified in patients with IE. Patients with IE had higher odds of in‐hospital mortality, shock, and ICU admission.

We acknowledge the following limitations of our study. We could not obtain access to the immunosuppressive regimen for LT recipients. We could not ascertain if the etiological organism would vary based on differences in the regimen. We could not identify causative organisms in 49% of cases of IE with LT. We were also unable to assess the effect of the time from immunosuppressant induction on the distribution of organisms. Furthermore, NIS are administrative databases that rely on the accuracy of coders. In addition, they lack clinical information on lab investigations, imaging, pharmacotherapy, and compliance. Finally, NIS is a cross‐sectional database that collects data during a single admission. Despite these limitations, the strength of our study is its large sample size, which limits the possibility of bias, which in turn imparts added validity to our conclusions. Inference may favor association analysis, and further studies may be warranted to establish causation.

## 5 Conclusion

Staphylococcus was present in 18.6% versus 1.9%, followed by enterococcus in 12.8% versus 1.6%, streptococcus in 6.9% versus 0.7%, and fungal infections in 2.9% versus 0.3% in the IE with LT group compared with IE without LT. Concomitant IE and LT were associated with increased in‐hospital death, ICU stay, and shock. The IE group was also associated with increased LOS and total charges compared to the LT without IE. Our study provides comparative insights that underscore the need for early identification and tailored management by recognizing the unique clinical profiles among patients with IE and LT. Further research is needed to evaluate the characteristics of attributable organisms for IE in LT recipients.

## Conflicts of Interest

The authors declare no conflicts of interest.

## Supporting information


Supplementary Table I. Patient demographics and hospital characteristics stratified by development of Infective Endocarditis. Supplementary Table II.Underlying Comorbidities seen in patients stratified by development of Infective Endocarditis*‐ Values < 10 were not reported as per HCUP policy. **Supplementary Table III‐ Bacterial infections, stratified by the presence of Infective Endocarditis**.

## Data Availability

The data that support the findings of this study are available in National Inpatient Sample at https://hcup-us.ahrq.gov/nisoverview.jsp. These data were derived from the following resources available in the public domain: ‐ Health care Cost and Utilization Project, https://hcup-us.ahrq.gov/tech_assist/centdist.jsp.
