# Supplementary material for: The Prevalence and Characteristics of Infective Endocarditis in Liver Transplant Recipients: Insights From National Inpatient Sample Database
Source: Clin Cardiol. 2025 Apr 14;48(4):e70130. doi: 10.1002/clc.70130 (PMC11995030; doi:10.1002/clc.70130)
Supplement: Supplementary file 1 — Supplementary Table I. Patient demographics and hospital characteristics stratified by development of Infective Endocarditis. Supplementary Table II.Underlying Comorbidities seen in patients stratified by development of Infective Endocarditis*‐ Values < 10 were not reported as per HCUP policy. Supplementary Table III‐ Bacterial infections, stratified by the presence of Infective Endocarditis. [file CLC-48-e70130-s001.docx]

**Supplementary Table I. Patient demographics and hospital characteristics stratified by development of Infective Endocarditis**

| **Demographics** | **Absence of IE n(%)** | **Presence of IE n(%)** | **p-value** |
| --- | --- | --- | --- |
| **Age Category** |  |  | 0.09 |
| 18-44 | 2,250 (13.1) | 35 (6.9) |  |
| 45-64 | 75,615 (44.4) | 215 (42.2) |  |
| >65 | 72,275 (42.5) | 260 (51) |  |
| **Sex** |  |  | **0.03** |
| Male | 102,480 (60.2) | 360 (70.6) |  |
| Female | 67,660 (39.8) | 150 (29.4) |  |
| **Race** |  |  | 0.29 |
| White | 120,155 (70.6) | 365 (71.6) |  |
| African American | 15,605 (9.2) | 75 (14.7) |  |
| Hispanic | 23,570 (13.9) | 55 (10.8) |  |
| Asian/Pacific Islander | 4,645 (2.7) | ** |  |
| Native American | 1,215 (0.71) | 0 (0) |  |
| Other | 4,950 (2.90) | 10 (1.9) |  |
| **Primary expected payer** |  |  | 0.51 |
| Medicare | 104,170 (61.2) | 360 (70.6) |  |
| Medicaid | 17,680 (10.4) | 50 (9.8) |  |
| Private Insurance | 43,380 (25.5) | 95 (18.6) |  |
| Uninsured | 1,605 (.9) | 0 |  |
| **Median Household Income** |  |  | 0.74 |
| Lowest Quartile | 43,965 (25.8) | 140 (27.5) |  |
| Second Quartile | 44,470 (26.1) | 130 (25.5) |  |
| Third Quartile | 44,650 (26.2) | 150 (29.4) |  |
| Fourth Quartile | 37,055 (21.8) | 90 (17.7) |  |
| **Region of Hospital** |  |  | 0.36 |
| Northeast | 33,600 (19.8) | 85 (16.7) |  |
| Midwest | 37,035 (21.8) | 140 (27.5) |  |
| South | 65,905 (38.7) | 170 (33.3) |  |
| West | 33,600 (19.8) | 115 (22.6) |  |
| **Location of the hospital** |  |  | 0.50 |
| Rural location | 7,315 (4.3) | 15 (2.9) |  |
| Urban location | 162,825 (95.7) | 495 (97.1) |  |
| **Teaching status of the hospital** |  |  | 0.85 |
| Non-teaching hospitals | 3,485 (17.9) | 95 (18.6) |  |
| Teaching hospitals | 139,655 (82.1) | 415 (81.4) |  |
| **Bed size of Hospital** |  |  | 0.71 |
| Small | 21,385 (12.6) | 55 (10.8) |  |
| Medium | 3,305 (22.5) | 130 (25.5) |  |
| Large | 110,450 (64.9) | 325 (63.7) |  |

**Supplementary Table II.Underlying Comorbidities seen in patients stratified by development of Infective Endocarditis***- Values▒<▒10 were not reported as per HCUP policy

|  | **Absence of IE n(%)** | **Presence of IE n(%)** | **p-value** |
| --- | --- | --- | --- |
| Prosthetic Heart Valve | 2,330 (1.4) | 25 (4.9) | **0.002** |
| Acute myocardial infarction | 14,040 (8.3) | 60 (11.8) | 0.198 |
| Congestive heart failure | 34,545 (20.3) | 200 (39.2) | **< 0.001** |
| Peripheral Vascular Disease | 9,645 (5.7) | 65 (12.8) | **0.002** |
| Cerebrovascular disease | 9,065 (5.3) | 80 (15.7) | **< 0.001** |
| Dementia | 4,045 (2.4) | 15 (2.9) | 0.709 |
| Chronic obstructive pulmonary disease | 29,385 (17.3) | 105 (20.6) | 0.366 |
| Rheumatoid disease | 3,295 (1.9) | ** | 0.481 |
| Peptic ulcer disease | 2,855 (1.7) | 10 (2) | 0.824 |
| Mild liver disease | 157,350 (92.5) | 480 (94.1) | 0.531 |
| Diabetes without complications | 34,680 (20.4) | 130 (25.5) | 0.199 |
| Diabetes with complications | 51,620 (30.3) | 180 (35.3) | 0.265 |
| Hemiplegia or paraplegia | 2,240 (1.3) | 5 (1) | 0.765 |
| Renal disease | 97,295 (57.2) | 360 (70.6) | **0.005** |
| Cancer | 7,995 (4.7) | 20 (3.9) | 0.71 |
| Moderate to severe liver disease | 15,930 (9.4) | 35 (6.9) | 0.387 |
| Metastatic Cancer | 4,740 (2.8) | 15 (2.9) | 0.923 |
| AIDS | 415 (0.2) | 0 | 0.64 |
| Hyperlipidemia | 41,950 (24.7) | 130 (25.5) | 0.846 |
| Smoking | 60,790 (35.7) | 200 (39.2) | 0.472 |
| Alcohol abuse | 15,440 (9.1) | 20 (3.9) | 0.07 |
| Obesity | 20,000 (11.7) | 55 (10.8) | 0.759 |
| ALD | 6400 (3.8) | 10 (2) | 0.339 |
| Hepatitis B | 3330 (2) | 15 (2.9) | 0.473 |
| Hepatitis C | 16320 (9.6) | 55 (10.8) | 0.68 |
| NASH | 4970 (2.9) | 0 | 0.08 |
| Hepatocellular carcinoma | 1445 (0.8) | 10 (2) | 0.219 |
| Ascites | 11640 (6.8) | 45 (8.8) | 0.429 |
| Varices | 580 (0.3) | 0 | 0.55 |
| Spontaneous Bacterial Peritonitis | 820 (0.5) | 15 (2.9) | **<0.001** |
| Hepatorenal syndrome | 1660 (1) | 0 | 0.322 |
| Hepatic encephalopathy | 10805 (6.4) | 60 (11.8) | **0.026** |

**Supplementary Table III- Bacterial infections, stratified by the presence of Infective Endocarditis**

|  | **Absence of IE n(%)** | **Presence of IE n(%)** | **p-value** |
| --- | --- | --- | --- |
| Staphylococcus | 3195 (1.9) | 95 (18.6) | **<0.001** |
| Enterococcus | 2,665 (1.6) | 65 (12.8) | **<0.001** |
| Gram-negative bacteria | 14,085 (8.3) | 50 (9.8) | 0.57 |
| Streptococcus | 1270 (0.7) | 35 (6.9) | **<0.001** |
| Fungal infections | 540 (0.3) | 15 (2.9) | **<0.001** |
